# Effect of Adding Natural Inulin on the Quality of Beef Myofibrillar Protein Gels

**DOI:** 10.3390/polym18080966

**Published:** 2026-04-16

**Authors:** Xuchen Ji, Yanbin Wang, Chunqing Shi, Mengjie Zhang, Zhouya Bai, Chonghui Yue, Libo Wang, Peiyan Li, Denglin Luo, Sihai Han

**Affiliations:** 1 College of Food and Bioengineering, Henan University of Science and Technology, Luoyang 471023, China; jixuchen2023@163.com (X.J.);; 2 Henan Engineering Research Center of Food Raw Materials, Luoyang 471023, China; 3 International Joint Laboratory of Green Processing and Quality and Safety Control of Food of Henan Province, Henan University of Science and Technology, Luoyang 471023, China

**Keywords:** natural inulin, beef myofibrillar protein, gel quality, textural properties, water distribution

## Abstract

To investigate how natural inulin (FI) influences the quality of heat-induced beef myofibrillar protein (BMP) gels, BMP gel systems were prepared with graded FI concentrations (1%, 2%, 3%, 4%, and 5%). Texture analysis (TA), low-field nuclear magnetic resonance (LF-NMR), rheological measurements, scanning electron microscopy (SEM), and Fourier transform infrared spectroscopy (FT-IR) were used to systematically characterise changes in gel properties, water migration and distribution, microstructure, and protein secondary structure. The results showed that the improvement in gel quality produced by inulin was concentration-dependent. FI at addition levels of 1–2% promoted the ordered intermolecular cross-linking of beef myofibrillar proteins, thereby facilitating the formation of a homogeneous and compact three-dimensional gel network, as confirmed by SEM and CLSM observations. Notably, 2% FI was identified as the optimal addition level for the BMP gel system. Compared with the control group, this treatment produced the highest relative β-sheet content (82%) among all groups, optimised the internal water distribution of the gel by reducing the proportion of free water, enhanced the water-holding capacity of the gels (*p* < 0.05), and preserved the elasticity-dominated solid-state characteristics of the BMP gel system (tan δ < 1), indicating that FI improved gel strength without changing its fundamental properties. These findings provide an important theoretical basis and practical technical parameters for the development of functional beef products with both desirable texture and high dietary fibre content.

## 1. Introduction

Myofibrillar proteins are key functional components of meat products. Their thermally induced gelling ability determines product texture, water-holding capacity, and sensory quality and underpins the formation of three-dimensional network structures in emulsified sausages, restructured meat, and minced meat products [1,2]. As a high-quality source of animal protein, beef myofibrillar protein (BMP) plays an important role in the development of high-quality beef products. However, because of its specific protein composition and molecular conformation, the gel network formed by BMP under heat-induced conditions often shows defects such as insufficient strength and non-uniform water retention [3]. This problem is particularly evident in health-oriented processing systems, such as low-salt and low-fat formulations, where it can readily lead to loose product texture and increased juice loss, thereby limiting, to some extent, the research, development, and industrial production of clean-label beef products [4,5].

In recent years, the incorporation of polysaccharides into protein gel systems to regulate gel network structure and improve water-holding capacity and mechanical properties has become a major research focus in food colloid science [6,7,8]. Compared with traditional chemical additives, natural polysaccharides show strong potential for modifying animal protein gels because of their wide availability, high safety, and functional properties. Natural inulin (FI) is a plant-derived storage polysaccharide composed of fructose units linked by *β*-2,1 glycosidic bonds in a linear chain that typically terminates in a glucose residue. As a high-quality dietary fibre, inulin not only exerts physiological effects such as regulating the gut microbiota and improving blood glucose and lipid levels but also shows promise in food gel systems because of its excellent hydration and gelling properties. Previous studies have shown that the addition of inulin can prolong the gastrointestinal retention time of drugs in gel systems and thereby enhance bioavailability [9]; the incorporation of FI into pork sausages can effectively improve product texture and storage stability [10]; and, in poultry protein gel systems, FI has also been reported to strengthen network structure and reduce cooking loss [11].

However, most existing studies have focused on the application of FI in pork or poultry myofibrillar protein gel systems, whereas systematic investigations of the interaction mechanism between FI and beef myofibrillar protein (BMP) during heat-induced gelation remain limited. In particular, the regulatory effects of FI on BMP denaturation and aggregation behaviour, microstructural evolution, and water distribution dynamics during heat-induced gelation have not yet been systematically elucidated. Accordingly, this study used BMP as the research object and prepared heat-induced gels containing different mass fractions of FI (1%, 2%, 3%, 4%, and 5%). Rheological testing, texture analysis (TA), low-field nuclear magnetic resonance (LF-NMR), scanning electron microscopy (SEM), and Fourier transform infrared spectroscopy (FT-IR) were comprehensively applied to investigate the effects of FI addition level on BMP gel properties and to further reveal the underlying mechanism, thereby providing a theoretical basis and technical support for the development of high-fibre, low-fat functional beef products.

## 2. Materials and Methods

### 2.1. Instrumentation

TA.XT Plus texture analyser (fitted with a P/50 cylindrical probe; Stable Micro Systems, Godalming, Surrey, GU7 1YL, UK); Niumag Micro MR 20025 LF-NMR instrument (proton resonance frequency 18.17 MHz; Suzhou Niumag Electronics Technology Co., Ltd., Suzhou, China); Nikon ECLIPSE Ti-E inverted laser confocal microscope (equipped with an A1 R laser; Nikon Corporation, Tokyo, Japan); MCR 92 rheometer (Anton Paar, Graz, Austria); JSM-6360LV scanning electron microscope (JEOL Ltd., Tokyo, Japan); Nicolet iS50 FT-IR spectrometer (Thermo Fisher Scientific, Waltham, MA, USA); Mars 6240/50 variable-frequency microwave oven (CEM Corporation, Stallings, NC, USA).

### 2.2. Materials and Reagents

Fresh beef Longissimus thoracis-et-lumborum muscle was obtained from 24-month-old Simmental cattle. According to the supermarket supervisor, the carcasses underwent electrical tenderisation during slaughter to accelerate rigor mortis and preliminary ageing. The meat was purchased from XX Supermarket within 4 h of slaughter; no additional carcass ageing was performed, and visible fat and connective tissue were removed. Samples were stored frozen at −20 °C and thawed for use within 72 h. Natural inulin (degree of polymerisation 10–30, purity ≥ 98%, Sensus, Arnhem, The Netherlands, grade GR) was used. NaCl, Tris, EDTA, and phosphate (analytical grade) were obtained from Sinopharm Chemical Reagents Co., Ltd., Shanghai, China. Nile Red (biological staining grade) was obtained from Shanghai Yuanye Biotechnology Co., Ltd., Shanghai, China. All other reagents were of analytical grade.

### 2.3. Extraction of Myofibrillar Proteins and Preparation of Gels

#### 2.3.1. Extraction of Myofibrillar Proteins

**Figure 1:**
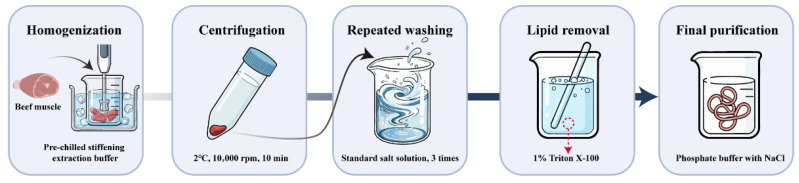
Flowchart of myofibrillar protein extraction.

Beef myofibrillar protein (BMP) was extracted according to the method of Liu et al. [12]. Thawed beef longissimus dorsi muscle was trimmed of visible connective tissue and fat and then minced. Pre-chilled stiffening extraction buffer (100 mmol/L Tris, 10 mmol/L EDTA, pH 7.0) was added at a solid-to-liquid ratio of 1:4 (g: mL), and the mixture was homogenised for 2 min at 10,000 rpm in an ice-water bath. The homogenate was centrifuged at 10,000 rpm for 10 min at 2 °C, after which the supernatant was discarded. The pellet was then resuspended in standard salt solution (100 mmol/L KCl, 1 mmol/L EGTA, 2 mmol/L MgCl_2_, 10 mmol/L K_2_HPO_4_/KH_2_PO_4_, 1 mmol/L NaN_3_, pH 7.0) at the same solid-to-liquid ratio, homogenised again, centrifuged, and the supernatant discarded. This washing procedure was repeated three times. The extraction was then repeated twice using a standard salt solution containing 1% Triton X-100 to remove membrane lipid components. Finally, the pellet was washed once with standard salt solution, and the resulting pellet was collected as purified BMP. The extracted BMP was dissolved in 40 mmol/L phosphate buffer (pH 7.0) containing 0.5 mol/L NaCl, adjusted to a protein concentration of 70 mg/mL, and stored at 4 °C until use. The process flowchart for myofibrillar protein extraction is shown in Figure 1.

#### 2.3.2. Preparation of FI-BMP Gel

For the preparation of the FI-BMP gels, 5 mL aliquots of BMP solution were mixed with FI to give final concentrations of 1–5% (*w*/*v*) [12]. Each mixture was magnetically stirred for 1 h to ensure complete dissolution, followed by equilibration at 20 °C for 10 min. Gelation was then induced by sequential microwave-assisted heating: samples were first heated to 48 °C at 150 W and held for 5 min, after which the temperature was raised to 68 °C at 350 W and maintained for 1 min. Immediately after heating, the samples were transferred to an ice-water bath and chilled for 30 min and were then allowed to stand at ambient temperature for 2 h. FI-BMP gels with graded FI concentrations were thus obtained.

### 2.4. Texture Characteristics of the Gel

Texture properties of the gel samples were measured using a texture analyser according to Zhang et al. [13], with slight modifications. The operating parameters were as follows: pre-test speed, 1.0 mm/s; test speed, 0.5 mm/s; post-test speed, 1.0 mm/s; compression depth, 50% of sample height; interval between the two compressions, 5 s; and trigger force, 5.0 g. During measurement, each gel sample was placed at the centre of the platform, and the probe was aligned vertically with the midpoint of the sample to minimise edge effects. Hardness, elasticity, cohesiveness, adhesiveness, and chewiness were recorded. Each sample was tested three times, and the mean values were used for subsequent statistical analysis.

### 2.5. Determination of the Moisture Distribution Characteristics of Gels

Low-field nuclear magnetic resonance (LF-NMR) was used to evaluate water distribution and mobility within the gel samples following Yi et al. [14], with minor modifications. For each measurement, approximately 2.5 g of gel was accurately weighed and transferred to an NMR glass tube with an internal diameter of 25 mm. The transverse relaxation time (T_2_) was determined using the Carr–Purcell–Meiboom–Gill (CPMG) pulse sequence. Instrument settings were as follows: 90° pulse width (P90), 5.50 μs; 180° pulse width (P180), 11.00 μs; sampling bandwidth (SW), 200 kHz; delay time (D3), 20 μs; and repetition time (TR), 2000 ms. Additional parameters were an analogue gain (RG1) of 20, a digital gain (RG2) of 3, 4 signal accumulations (NS), an echo time of 250 μs, and 10,000 acquired echoes. All samples were analysed in triplicate, and the mean values were used for subsequent interpretation.

### 2.6. Rheological Properties of Gels

Rheological properties of the gel samples were analysed using a rotational rheometer based on the method of Ghosh et al. [15]. The gap between the measuring plates was set to 1.00 mm. Before testing, samples were pre-sheared to eliminate any residual deformation history. For the dynamic temperature ramp test, a constant strain of 0.50% was applied, with frequency sweeps from 1 to 10 Hz. The temperature was increased from 25 °C to 80 °C at a rate of 2 °C/min. To prevent artefacts arising from moisture loss, the sample edges were sealed with a thin layer of silicone oil. The storage modulus (G′), loss modulus (G″), and loss tangent (tan *δ*) were continuously monitored and recorded throughout the test.

### 2.7. Confocal Laser Scanning Microscopy (CLSM)

Confocal laser scanning microscopy (CLSM) was used to visualise the microstructure of the gel samples, following Zhang et al. [16], with slight modifications. A representative portion of each gel was stained with Nile Red at a concentration of 0.005 g/L and incubated in the dark for 10 min to ensure uniform staining. The stained sample was placed on a glass slide, sealed with a coverslip, and observed under an inverted microscope. Excitation was provided by a 488 nm laser, and fluorescence emission was collected in the 500–530 nm range. Images were acquired using a 10× objective, with z-axis scanning performed at 5.72 μm intervals. Fifteen consecutive layers were collected, giving a total imaging depth of approximately 86 μm for 3D reconstruction. Three different fields of view were analysed for each sample to obtain representative images.

### 2.8. SEM Analysis of the Gel

The microstructure of freshly prepared gel samples was examined by scanning electron microscopy (SEM) according to a modified method of Zhuang et al. [17]. Briefly, gel samples were pre-frozen at −20 °C for 12 h on aluminium trays sealed with moisture-impermeable plastic film to minimise water loss and promote uniform ice-crystal formation. The frozen samples were then freeze-dried under vacuum at −50 °C for 48–72 h until fully dehydrated. After drying, fragments with clean, intact fracture surfaces were carefully selected, mounted on SEM stubs with conductive carbon tape, and sputter-coated with a thin gold layer (~10 nm) to improve conductivity and image contrast. High-resolution SEM images were acquired at an accelerating voltage of 5.0 kV, focusing on representative cross-sectional areas to characterise pore architecture, surface topography, and network homogeneity.

### 2.9. FT-IR Analysis of the Gel

FT-IR spectroscopy was used to evaluate changes in the secondary structure of the gel samples following Wu et al. [18], with slight modifications. Approximately 2.0 mg of lyophilised gel was accurately weighed and mixed with anhydrous potassium bromide (KBr) at a mass ratio of 1:100. The mixture was thoroughly ground in a mortar until uniform and then compressed at 18 MPa for 30 s to form a transparent pellet. Spectra were collected on a Fourier transform infrared spectrometer over the range 4000–400 cm^−1^ at a resolution of 4 cm^−1^ with 32 scans. Background correction was performed with a pure KBr pellet before sample measurement. The spectra were analysed using OMNIC 9.2.86 software, with particular attention to the amide I region (1600–1700 cm^−1^), which was subjected to deconvolution and curve-fitting to quantify the relative proportions of different secondary structural elements.

### 2.10. Data Processing

All measurements were performed in triplicate (n = 3). Data were analysed using IBM SPSS Statistics 27.0 (IBM Corp., Armonk, NY, USA). Continuous variables are presented as mean ± standard deviation (SD). Statistical significance was evaluated using the Waller–Duncan k-ratio *t* test for multiple comparisons and independent-sample *t*-tests for pairwise comparisons, with *p* < 0.05 considered statistically significant. Figures were prepared using GraphPad Prism 9.5 (GraphPad Software, LLC, Boston, MA, USA) and OriginPro 2024 (OriginLab Corporation, Northampton, MA, USA).

## 3. Results and Discussion

### 3.1. Textural Analysis

**Figure 2:**
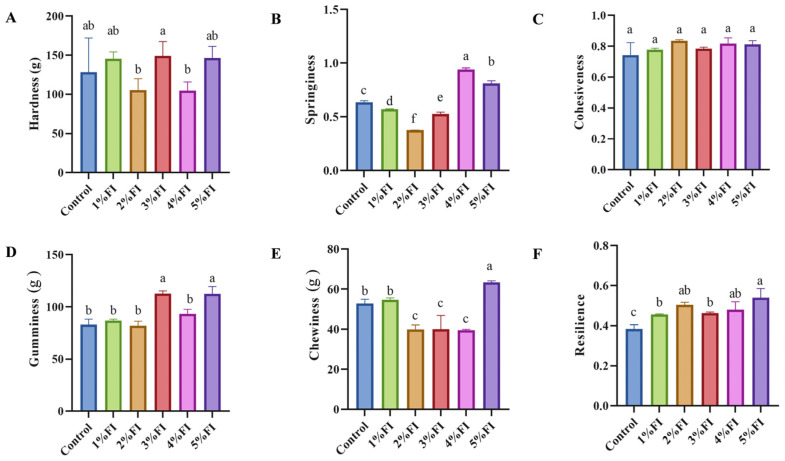
Effects of different levels of FI addition on the texture properties of BMP gels: (**A**) hardness; (**B**) springiness; (**C**) cohesiveness; (**D**) gumminess; (**E**) chewiness; (**F**) resilience. Different superscript letters above the bars indicate significant differences among groups at *p* < 0.05.

To evaluate the effect of FI addition on gel texture, the hardness, elasticity, and related parameters of the beef protein gels were measured using a texture analyser, and the results are presented in Figure 2. Although the substantial standard deviation observed in the control group limited clear statistical differentiation (as indicated by the significance markers), the overall textural profile demonstrates that FI content exerted a pronounced effect on the gel properties. Relative to the control, the 2% FI group did not show the greatest gel hardness, and its hardness was similar to that of the 4% FI group. Hardness reached its maximum in the 3% FI group and then declined significantly in the 4% FI group, which may be attributable to the loosening of the protein network caused by FI over-hydration at concentrations of ≥3%. When FI addition was further increased to 5%, hardness showed a slight recovery, possibly because FI promoted the formation of a composite network through hydrogen bonding and hydrophobic interactions. This observation agrees with Luo et al. [19], who reported that an appropriate amount of FI (3%) can significantly increase gel hardness. Elasticity showed a decreasing-then-increasing trend with rising FI content and reached its lowest value in the 2% FI group. Although the 3–5% FI groups showed partial recovery of elasticity through disulphide cross-linking, elasticity did not return to the level of the control group. Wang et al. [20], in their study of yak myofibrillar protein gels, found that small molecules can regulate protein conformational unfolding and promote the conversion of thiol groups into disulphide bonds, thereby forming a more uniform and compact gel network. This mechanism is similar to that observed here, where 3–5% FI appeared to restore gel elasticity through disulphide-bond cross-linking.

However, Guo et al. [21], in their study of FI with different degrees of polymerisation in wheat gluten protein gels, reported that FI inhibits intermolecular cross-linking of protein molecules through steric hindrance and significantly reduces disulphide-bond content in the gel. This suggests that plant and animal proteins respond differently to FI [12]; in the present study, disulphide bonds in animal proteins appeared more susceptible to FI-mediated cleavage and rearrangement. It may therefore be inferred that an appropriate amount of FI promotes favourable transformations of disulphide bonds, whereas FI supplementation at concentrations of ≥3% may induce non-specific and disordered protein aggregation, which is associated with disruption of interprotein interactions. Huang et al. [22] reported that FI can form a dense composite network with myofibrillar proteins through hydrogen bonding, thereby significantly reducing protein surface hydrophobicity. This provides theoretical support for explaining why high FI concentrations led to reduced gel hardness in the present study.

### 3.2. Analysis of Moisture Distribution

**Figure 3:**
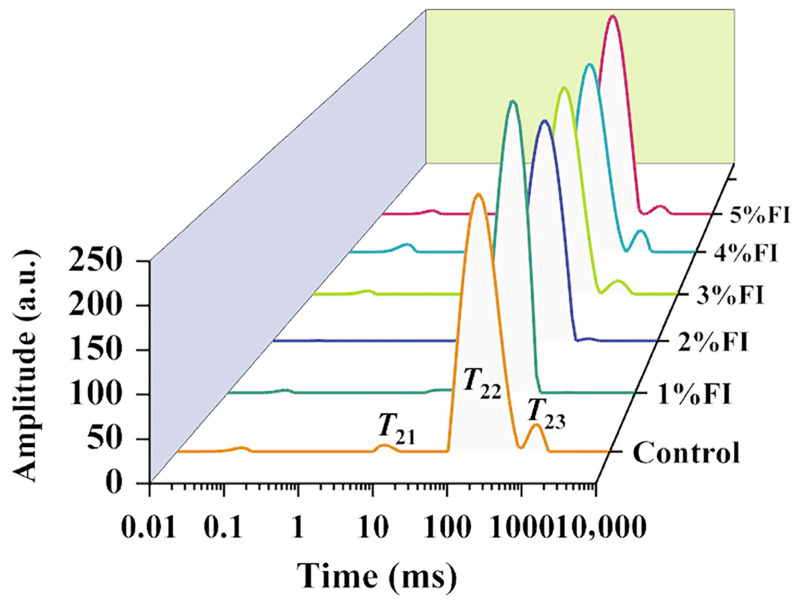
Inversion plot of the LF-NMR data for the gel.

LF-NMR inversion spectra of relaxation times are shown in Figure 3, and the corresponding water distribution parameters are summarised in Table 1. As indicated by Figure 3 and Table 1, different water fractions responded differently to FI addition, with free water (*T*_23_) showing the greatest sensitivity. Compared with the control, the relative content of free water (*A*_23_) decreased significantly in the 1% and 2% FI groups, accompanied by a corresponding increase in immobilised water (*A*_22_). This suggests that, owing to its strong hydration capacity, low concentrations of FI (1–2%) can adsorb free water and promote its conversion to bound water (*T*_22_), thereby altering water distribution within the system. In addition to the changes in water content, clear shifts were observed in the peak positions of *T*_23_ (free water) and *T*_21_ (tightly bound water) in both the 1% and 2% FI groups, whereas the peak position of *T*_22_ (immobilised water) exhibited negligible variation. This further confirms that low-concentration FI can regulate the distribution of different water states in the system through hydration interactions. This finding is consistent with a previous study of fish paste gels [23], which suggested that changes in water content are primarily associated with intermolecular forces between water molecules and proteins.

The content of tightly bound water *A*_21_ increased when moderate amounts of FI were added, showing an overall trend of first decreasing and then increasing. This may be because low levels of FI primarily enhance the water-binding ability of the protein, which is mainly reflected in an increase in weakly bound water, whereas FI at addition levels of ≥3% increases tightly bound water because FI competes for water molecules and binds them more strongly. Bao et al. [24] found that dietary fibre can regulate transformations in protein secondary structure during heat treatment through steric hindrance and intermolecular hydrogen bonding; at low concentrations, it promotes ordered protein cross-linking, whereas at high concentrations it interferes with the formation of *β*-sheet structures. This is consistent with the pattern by which inulin regulates the secondary structure of BMP in the present study. Changes in the *T*_22_ value of non-free water further support this hydration-regulating effect; the increase in *T*_22_ in the 2% FI group suggests that, after FI filled voids within the molecular network, the intermolecular hydration environment was improved. Therefore, the hydrophilic colloidal properties of inulin, by reducing free water and strengthening water control, synergistically enhance intermolecular interactions.

### 3.3. Analysis of Rheological Properties

**Figure 4:**
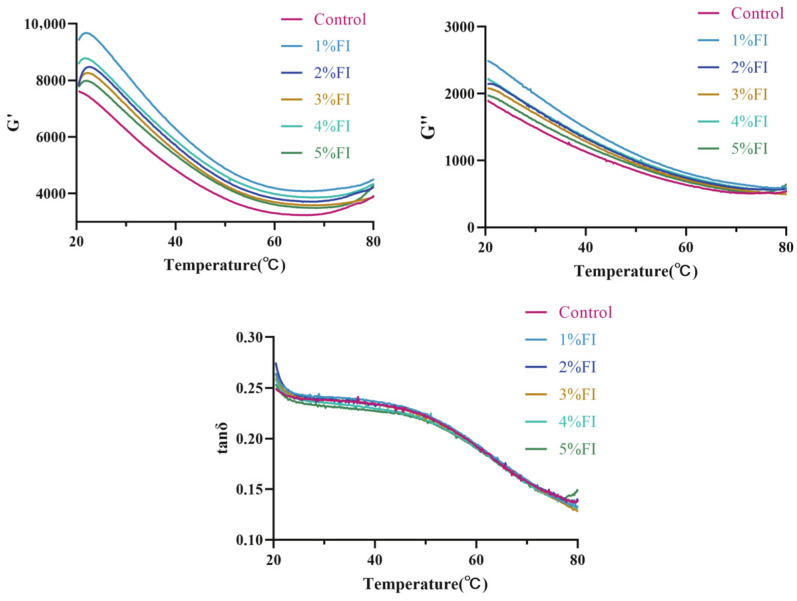
Graph of rheological results.

Rheological analysis (Figure 4) showed that FI addition affected the viscoelastic properties of the BMP gels. Although the overall evolution trends of the samples were generally similar, the sample with 1% FI exhibited distinctly higher values of both storage modulus (G′) and loss modulus (G″) than the control group during the entire heating process. This result indicates that an appropriate amount of natural inulin can enhance both the elastic framework and viscous dissipation capacity of the gel network through intermolecular hydrogen bonding and hydrophobic interactions, thereby improving the overall resistance of the system to deformation. This phenomenon is highly consistent with the findings of Sun et al. [25], who concluded that inulin can simultaneously enhance the elasticity and viscosity of protein gels, confirming that inulin can act as a structural enhancer during protein network formation at low concentrations.

As the temperature increased from 20 to 80 °C, G′ and G″ in all samples exhibited a two-stage pattern: a continuous and pronounced decrease from 20 °C to approximately 60 °C, followed by a relatively stable plateau from 60 °C to 80 °C. This indicates that heating disrupted the integrity of the gel network, causing simultaneous weakening of elasticity and viscosity and a gradual shift of the system towards more fluid-like behaviour. This trend is consistent with the findings of Tang et al. [26] on high-temperature-induced protein denaturation and degradation, which affect gel quality, suggesting that thermal treatment exerts a generally destructive effect on protein gel networks and that the addition of inulin did not alter this thermodynamic response.

When the inulin content reached or exceeded 2%, G′ and G″ continued to decrease as concentration increased, showing a concentration-dependent decay. One possible explanation is that, at high concentrations, inulin molecules linked by *β*-2,1-glycosidic bonds tend to aggregate between chains and form local clusters. These clusters may act as structural defects that interrupt the continuity of cross-linking within the protein gel matrix. At the same time, FI at addition levels of ≥3% occupied substantial space within the system, and the resulting steric hindrance interfered with the ordered cross-linking and hydrophobic aggregation of protein molecules.

As suggested by Han et al. [27], the sensitivity of gel systems to steric hindrance is closely related to temperature, and heating may intensify this adverse effect, which provides theoretical support for the behaviour observed in the present study. Although G′ and G″ changed with inulin concentration and temperature, the loss tangent (tan *δ*) remained below 1 in all groups, indicating that inulin addition did not alter the elasticity-dominated solid nature of the gels but merely modulated their mechanical strength in a concentration-dependent manner. This result is similar to the conclusion of Toczek et al. [28], who reported that chitosan, as a polysaccharide, primarily regulates the mechanical performance of protein-based gels rather than changing their fundamental viscoelastic characteristics.

### 3.4. CLSM Analysis

**Figure 5:**
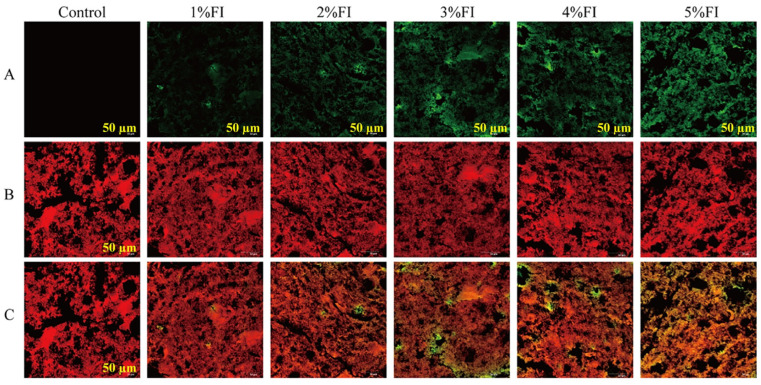
Images of CLSM results: (**A**) green channel image (representing polysaccharides); (**B**) red channel image (representing protein structures); (**C**) merged channel image.

CLSM images of the gels are presented in Figure 5. In Figure 5, Panels A, B, and C show the fluorescence distribution of FI (green), beef protein (red), and the merged image, respectively; the black areas in all panels represent water-filled pores or voids within the gel network.

The control gel displayed a relatively loose and disordered protein network (red fluorescence, Panel B), and large, irregular voids were clearly visible in both the protein and merged images. In the 1% FI group, both the size and number of voids were markedly reduced. The green fluorescence corresponding to FI was mainly distributed along the protein network framework, indicating that FI had been incorporated into the protein matrix and thereby contributed to strengthening the network structure. As FI concentration increased further (≥3%), the voids within the protein network gradually became larger, and the protein matrix showed a more aggregated morphology, as evidenced by the uneven distribution of red fluorescence and the expansion of black regions in the merged images (Panel C). Co-localisation of FI (green) and protein (red), appearing as yellow/orange fluorescence in Panel C, became more obvious as the FI concentration increased from 3% to 5%, indicating stronger interactions and aggregation between FI and protein molecules. Hou et al. [29], in their study of pea protein–polysaccharide composite emulsion gels, reported that protein–polysaccharide interactions regulate the aggregation state of proteins. Moderate aggregation favours the formation of a continuous and dense network, whereas high polysaccharide concentrations induce microphase separation, resulting in disordered protein aggregation, heterogeneity, and uneven porosity within the gel network. This is highly consistent with the present results: low FI addition (1%) induced moderate protein aggregation and reduced pore size, whereas excessive FI (≥3%) triggered microphase separation, leading to enlarged voids and aggregated protein networks. Lv et al. [30], in their study of egg protein–polysaccharide interactions, pointed out that this aggregation behaviour mainly involves protein conformational unfolding and changes in intermolecular forces, including hydrogen bonding, hydrophobic interactions, and disulphide cross-linking. CLSM observations in the present study showed that FI at addition levels of ≥3%, by associating with the protein structure, altered hydrophobic interactions between proteins and ultimately resulted in non-uniform aggregation. Chen et al. [31], in a CLSM study of whey protein-based emulsions, likewise found that inulin supplementation at concentrations of ≥3% disrupted emulsion structure, which corroborates the present findings.

### 3.5. SEM Analysis

**Figure 6:**
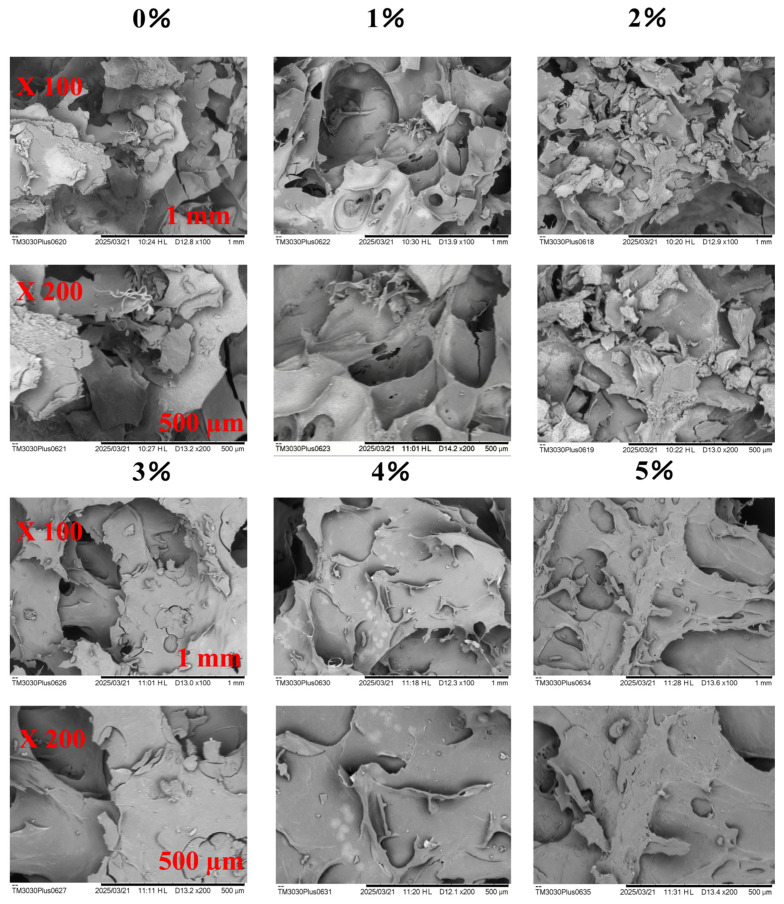
Scanning electron microscopy images.

SEM images of the freeze-dried gels are shown in Figure 6. Observation of the effects of different natural inulin (FI) loading levels on gel microstructure revealed that the 1% and 2% FI groups exhibited a more uniform and compact network, whereas the groups containing 3% FI or more showed pronounced variation in pore size together with the formation of coarse aggregates. At low concentrations (1–2%), inulin appears to promote ordered cross-linking by guiding the oriented exposure of active protein groups through intermolecular hydrogen bonding and hydrophobic interactions. At the same time, it may act as a spatial stabiliser by regulating the phase-separation balance between the protein phase and solvent phase, thereby preventing random spontaneous aggregation and promoting moderate aggregation that forms uniform pores. When FI was added at concentrations of ≥3%, the high polysaccharide loading in the BMP gel system induced non-specific and disordered over-aggregation of protein molecules; alternatively, the polysaccharides themselves may have aggregated and competitively occupied space, thereby disrupting the original protein network. Under these conditions, proteins shifted from oriented cross-linking to random aggregation, causing structural collapse and ultimately producing non-uniform coarse aggregates and a fragmented network. Therefore, a low inulin concentration is more favourable for regulating moderate protein aggregation and generating a superior gel microstructure.

Min [32] suggested that polysaccharides enhance gel-network stability by promoting hydrogen bonding among starch, proteins, and polysaccharides through steric repulsion and also noted that microstructure is directly related to gel functional properties. Tang [33] proposed that neutral polysaccharides regulate structure mainly through steric hindrance; as inulin is a neutral polysaccharide, its mechanism of action in the present system should likewise be dominated by steric effects. Zhao et al. [34] investigated the effect of inulin on yoghurt microstructure and concluded that the improvement mainly resulted from the abundance of hydrophilic groups in inulin, which increased both the strength and number of hydrogen bonds within the gel network. This interpretation is consistent with our results.

### 3.6. FT-IR and Secondary Structure Analysis

**Figure 7:**
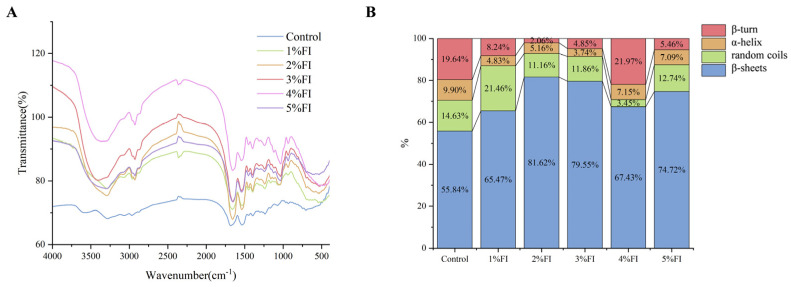
FT-IR spectrum (**A**) and proportion (**B**) of secondary structural components.

FT-IR was used to analyse the effects of different inulin addition levels on the molecular structure of the protein gels, and the results are shown in Figure 7A. At low addition levels (1% and 2% inulin), the O-H/N-H stretching band in the 3000–3600 cm^−1^ region became broader and more intense, indicating the formation of hydrogen bonds between hydroxyl groups in the inulin molecules and polar groups in the protein. In the 1500–1700 cm^−1^ region, the amide I band (~1650 cm^−1^) showed a sharper profile and a narrower half-width, while the amide II band (~1540 cm^−1^) exhibited a red shift, reflecting an increase in *β*-sheet content and more ordered molecular cross-linking within the protein secondary structure. In the 1000–1200 cm^−1^ region, coupling between the C–O–C stretching vibrations of polysaccharides and those of proteins was observed, further confirming the interaction between inulin and protein molecules. At high addition levels (3–5% FI), the band in the 3000–3600 cm^−1^ region became narrower and split, whereas the amide band in the 1500–1700 cm^−1^ region broadened and shifted towards higher wavenumbers, and the characteristic polysaccharide band in the 1000–1200 cm^−1^ region became distinct and sharp. These changes suggest that FI at addition levels of ≥3% may induce molecular self-aggregation, disrupt the hydrogen-bonding network within the gel system, and thereby disorder the protein secondary structure.

Further curve-fitting of the amide I band was performed to quantify the proportions of secondary-structure elements, and the results are shown in Figure 7B. The *β*-sheet content first increased and then decreased as inulin addition increased, reaching a maximum value of 32.1% in the 2% FI group. Because *β*-sheets are generally positively correlated with gel strength, a higher *β*-sheet content indicates a more stable gel network [35]. Conversely, the random coil content remained relatively low in the 2% FI group, whereas *β*-turn content showed a trend of first decreasing and then increasing. These results suggest that an appropriate amount of inulin (2%) promotes a shift in protein secondary structure towards a more ordered state and reduces the proportion of disordered conformations. Changes in α-helix content were accompanied by increases or decreases in *β*-folds or random coils. Nawrocka et al. [36] suggested that natural inulin influences α-helix content by increasing the degree of protein-chain unfolding and thereby regulating secondary structure. In a study of the effects of inulin on gluten proteins, Mu et al. [37] found that α-helices contribute to the maintenance of dough elasticity and hardness; this differs from the present results and may reflect structural and response differences between animal-derived and plant-derived proteins. Overall, 2% inulin was the most favourable level for inducing an ordered BMP secondary structure dominated by *β*-folds, thereby providing a molecular basis for the formation of a stable gel network.

## 4. Conclusions

This study investigated the effects of different FI levels on the properties of thermally induced BMP gels. The results show that the improvement in gel quality caused by inulin is concentration-dependent. Inulin at addition levels of 1–2% significantly promoted ordered protein cross-linking and produced a uniform, compact three-dimensional network structure. Among all treatments, 2% FI was identified as the optimal addition level, increasing gel hardness by 31.2% and elasticity by 20.1%, while significantly increasing *β*-sheet content, optimising water distribution, and enhancing water-holding capacity. By contrast, FI at addition levels of ≥3% disrupted protein-network continuity through self-aggregation and overhydration, leading to deterioration in gel texture and the appearance of coarse aggregates in the microstructure. Rheological results further showed that all gels retained elasticity-dominated solid-state characteristics (tan *δ* < 1), indicating that inulin improved gel strength without altering the fundamental nature of the system. These findings provide an important theoretical basis and practical technical parameters for the development of functional beef products that combine desirable texture with high dietary fibre content.

## Figures and Tables

**Table 1:** Effects of different FI additions on LF-NMR parameters of BMP gels.

Group	LF-NMR Parameters					
	*A*_21_/%	*A*_22_/%	*A*_23_/%	*T*_21_/ms	*T*_22_/ms	*T*_23_/ms
Control	1.535 ± 0.04 ^c^	94.47 ± 0.01 ^d^	3.95 ± 0.04 ^b^	7.06 ± 0.26 ^d^	151.99 ± 0.27 ^b^	932.60 ± 5.38 ^b^
1%FI	1.41 ± 0.03 ^d^	98.56 ± 0.04 ^a^	0.03 ± 0.01 ^f^	18.74 ± 0.45 ^a^	151.99 ± 0.29 ^b^	1072.27 ± 5.43 ^a^
2%FI	1.06 ± 0.05 ^f^	98.52 ± 0.15 ^a^	0.34 ± 0.01 ^e^	12.33 ± 0.38 ^b^	174.75 ± 0.33 ^a^	811.13 ± 6.36 ^c^
3%FI	1.26 ± 0.02 ^e^	95.38 ± 0.09 ^c^	3.31 ± 0.01 ^c^	5.34 ± 0.23 ^f^	132.19 ± 0.31 ^c^	1072.27 ± 3.41 ^a^
4%FI	2.53 ± 0.04 ^a^	92.79 ± 0.15 ^e^	4.49 ± 0.17 ^a^	6.14 ± 0.27 ^e^	151.99 ± 0.26 ^b^	1072.27 ± 5.41 ^a^
5%FI	1.71 ± 0.04 ^b^	96.55 ± 0.04 ^b^	1.71 ± 0.06 ^d^	9.33 ± 0.32 ^c^	151.99 ± 0.24 ^b^	1072.27 ± 2.77 ^a^

Data are presented as mean ± standard deviation (SD) of triplicate determinations (n = 3); different superscript letters within the same row indicate significant differences at *p* < 0.05.

## Data Availability

The data presented in this study are available upon request from the corresponding author.
